# Influence of ultrasound transmit frequency on measurement of global longitudinal strain on 2D speckle tracking echocardiography

**DOI:** 10.1038/s41598-023-49664-3

**Published:** 2023-12-14

**Authors:** Katsuomi Iwakura, Toshinari Onishi, Yasushi Koyama, Mutsumi Iwamoto, Satoshi Watanabe, Koji Tanaka, Yuko Hirao, Nobuaki Tanaka, Akinori Sumiyoshi, Masato Okada, Kota Tanaka, Shinichi Harada, Heitaro Watanabe, Atsunori Okamura

**Affiliations:** 1 Division of Cardiology, Sakurabashi Watanabe Hospital, 2-4-32, Umeda, Kita-ku, Osaka, Osaka 5300001 Japan; 2 Department of Cardiovascular Medicine, Sakai City Medical Center, 1-1-1 Ebaraji-cho, Nishi-ku, Sakai, Osaka 5938304 Japan

**Keywords:** Cardiology, Medical imaging

## Abstract

The reproducibility of longitudinal strain measured by 2D speckle tracking echocardiography (2DSTE) may be affected by ultrasound settings. This study investigated the effect of transmit ultrasound frequency on global longitudinal strain (GLS) by 2DSTE. Apical, 2- and 4-chamber, and long-axis views were obtained in consecutive 162 patients using Philips ultrasound devices. Three different frequency presets were used sequentially: high resolution (HRES, 1.9 to 2.1 MHz), general (HGEN, 1.6 to 1.8 MHz), and penetration mode (HPEN, 1.3 to 1.6 MHz). GLS values were determined for each preset using the Philips Q-station software, resulting in GLS-HRES, GLS-HGEN, and GLS-HPEN. Among the 151 patients with successfully measured GLS, a significant difference in GLS was observed among the three presets (*p* < 0.0001). GLS-HRES (− 17.9 ± 4.4%) showed a slightly smaller magnitude compared to GLS-HGEN (− 18.8 ± 4.5%, *p* < 0.0001) and GLS-HPEN (− 18.8 ± 4.5%, *p* < 0.0001), with absolute differences of 1.1 ± 1.0% and 1.1 ± 1.2%, respectively. This variation in GLS with frequency was evident in patients with both optimal (n = 104) and suboptimal (n = 47) image quality and remained consistent regardless of ultrasound devices, ischemic etiology, or ejection fraction. In conclusion, ultrasound frequency had only a modest effect on GLS measurements. GLS may be reliably assessed in most cases regardless of the ultrasound frequency used.

## Introduction

Left ventricular ejection fraction (LVEF) is widely used to assess left ventricular (LV) systolic function, but its reproducibility is limited for several reasons, such as requirement of geometric assumptions and the difficulty of accurate delineation of LV endocardial border^1,2^. Global longitudinal strain (GLS) by 2D speckle tracking echocardiography (2DSTE) is more reproducible^3,4^, and more sensitive to change in LV systolic function^5–7^ than LVEF. GLS provides additional prognostic value over LVEF in asymptomatic general population^7,8^, and in patients with cardiovascular disease such as cardiomyopathy^9–11^, valvular heart disease^12–15^, and heart failure^16–19^. GLS measurement is recommended for quantitative analysis of LV systolic function in a standard echocardiography whenever possible^20^.

Strain measurement by 2DSTE, however, may be affected by technical conditions including image quality^21–25^^.^ GLS measured on poor quality images may be less accurate than that on good quality images^26–28^, whereas one study reported no relationship between image quality and reproducibility of GLS^29^.

In patients with poor image quality due to pulmonary disease or obesity, low ultrasound frequency is beneficial to improve image quality^30^. Lower ultrasound transmit frequency is associated higher tissue penetration though image resolution might be reduced^30^. It is recommended that operators should start echocardiography study with a high transmit frequency and then adjust to lower frequencies if additional penetration of the sound wave is needed^30^. It is not clear whether the ultrasound frequency may affect the GLS measurement. We investigated the influence of ultrasound frequency on GLS magnitude and its association with image quality.

## Methods

### Study population

For the present study, we prospectively enrolled 162 consecutive patients who underwent 2D echocardiography in our echo lab from June to September in 2017. We excluded patients (1) who had arrhythmia affecting GLS measurement, such as atrial fibrillation and multiple extra beats, at the time of examination (2) who had too poor image quality to measure longitudinal strain (3) in whom proper speckle tracking could not be achieved after several attempts due to technical reasons. We did not exclude patients showing suboptimal image quality if proper speckle tracking was performed.

This study was conducted in accordance with the Declaration of Helsinki. The study protocol was approved by the hospital’s Ethics Committee. One of the investigators obtained the informed consent from each patient before the study.

### Echocardiography examination

2D echocardiography was performed using ultrasound devices by Philips (Andover, MA USA) including IE33, Affiniti 70G or EPIQ 7. IE33 and Affiniti were equipped with a S5-1 transducer, and EPIQ with a X5-1 transducer. We used tissue harmonic imaging for image acquisition, and the mean framerate was 64 [61, 68] frame per second. These devices had three presets of the fundamental transmission frequency to optimize 2D image quality, which could be easily changed during the examination. These presets were designated as (1) HRES, higher frequency for better image resolution; 2.1 MHz for IE33, 2.4 MHz for Affiniti, and 1.9 MHz for EPIQ, (2) HPEN, lower frequency for better penetration; 1.4 MHz for IE33, 1.6 MHz for Affiniti, and 1.3 MHz for EPIQ, (3) HGEN, optimizing the balance between HRES and HPEN settings for general imaging needs; 1.7 MHz for IE33, 1.8 MHz for Affiniti, and 1.6 MHz for EPIQ (preceding “H” is for harmonic imaging).

**Figure 1:**
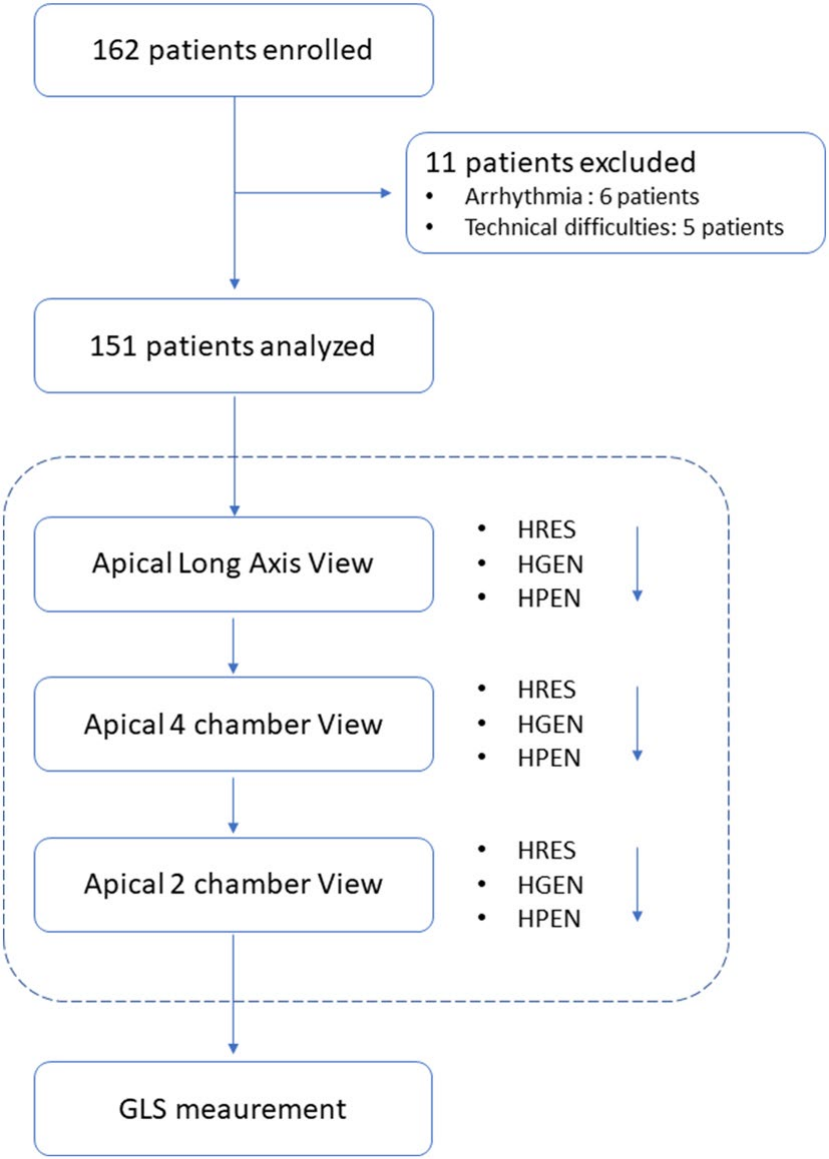
Enrollment and global longitudinal strain measurement. We enrolled consecutive 162 patients who underwent 2D echocardiography from June to September in 2017. Patients in whom speckle tracking was not successfully performed due to arrhythmia or image quality were excluded. Images from apical long-axis, 2-chamber and 4-chamber view were acquired sequentially using the HRES (higher frequency preset for better resolution), HGEN (intermediate setting between HRES and HPEN) and HPEN (lower frequency for better penetration) presets.

We acquired images from apical long-axis, 2-chamber and 4-chamber view using each frequency presets. In each apical view, we first acquired an image using the HRES preset during two cardiac cycles. We subsequently acquired the images using the HGEN preset, and then using the HPEN preset, without interruption (Fig. 1). We kept the position of a transducer as stable as possible, and patients were instructed to hold breath through all image acquisition. Image quality in each patient was determined by an echocardiographer who performed examination using images recorded by the HGEN setting. Suboptimal image quality was defined as images in which endocardial border of more than 2 segments was not well differentiated. Patients were considered to have suboptimal image quality if at least one of the apical images was determined as suboptimal.

An experienced echocardiographer blinded to the clinical data measured segmental longitudinal strain on stored images using Q-station software version 3.7 (Philips, Andover MA). At first, the end-systolic frame was defined in the apical long-axis view. Aortic valve closure was marked, and the Q-station software measured the time interval between the R-wave and aortic valve closure, which was used as a reference for the other view loops. We manually defined the mitral annulus and left ventricular apex with 3 index points at the end-systolic frame in each apical image. The Q-station software automatically traced 3 concentric lines on the endocardial border, mid-myocardial layer, and epicardial border, and followed the endocardium from this single frame throughout the cardiac cycle. If the automated tracking was inappropriate by visual assessment, we manually adjusted the region of interest. LV in each apical image was divided into 6 segments, and the tracking quality for each segment was validated by the operator. The software measured the percent of wall lengthening and shortening and calculated peak systolic longitudinal strain for each segment, and the results from all 3 planes were combined into a single bull’s-eye plot based on a 17-segment model. GLS was automatically calculated as an averaged value of peak longitudinal strain in all segments. GLS was measured twice in each group of images and averaged values were used for the statistical analysis.

We measured GLS on images by the HRES, HGEN and HPEN preset separately, and designated them as GLS-HRES, GLS-HGEN and GLS-HPEN, respectively. The relative difference between GLS-HRES and other GLS values was calculated as [absolute difference between GLS-HRES and GLS-HGEN (or GLS-HPEN) divided by GLS-HRES] × 100 (%). To avoid the confusion, we used ‘%’ instead of ‘%’ for absolute difference in GLS in this report.

We measured LVEF using the Auto-EF function on Q-station from apical 4- and 2 chamber images based on Simpson’s rule, and LVEF < 50% was defined as impaired LVEF.

### Statistical analysis

Continuous values are expressed as mean ± standard deviation for normally distributed variables or median [interquartile range] for non-normally distributed variables. Categorical variables are expressed as absolute frequencies or relative percentages. We made comparisons by one-way analysis of variance (ANOVA) for continuous variables, and significance of difference was calculated with the Bonferroni test for post-hoc analysis. Categorical variables were compared with Fisher’s exact test. Correlation between continuous variables was evaluated using Pearson's correlation test, and correlation coefficients were compared using Z scores produced by Fisher’s r to z transformation. Agreement among GLS values by three frequency presets were evaluated by the Bland–Altman plot. We compared GLS values among HRES, HGEN and HPEN by repeated measure ANOVA with the Bonferroni test for post-hoc analysis. We performed mixed design ANOVA to investigate whether changes in GLS among frequency presets was affected by other factors. All statistical analyses were performed using R (The R Foundation for Statistical Computing, Vienna, Austria) with a graphical user interface EZR (Saitama Medical Center, Jichi Medical University, Saitama, Japan).

## Results

### Patients characteristics

Among 162 patients enrolled for the present study, 11 patients were excluded because adequate speckle tracking was not achieved due to frequent extra beats (6 cases) or very poor image quality (5 cases). The final study population consisted of 151 (93.2%) patients (age: 68 [55, 75] years, male gender: 117 patients (77.5%)). The major reason for the echocardiography was coronary artery disease (CAD) in 58 patients (38.4%), arrhythmia in 33 patients (21.9%), valvular heart disease in 32 patients (21.2%) and others in 12 patients (8.0%). Mean LVEF was 60.2 ± 9.6% and 24 patients (15.9%) had LVEF < 50%.

We evaluated the inter- and intraobserver variability of the GLS measurement in 10 randomly selected subjects with optimal image quality and another 10 subjects with suboptimal image quality using the HGEN setting. The inter-and intraobserver variance was 0.72% and 0.65% for those with optimal image quality and 0.86% and 0.78% for those with suboptimal image quality, respectively.

### GLS and ultrasound transmit frequency

**Figure 2:**
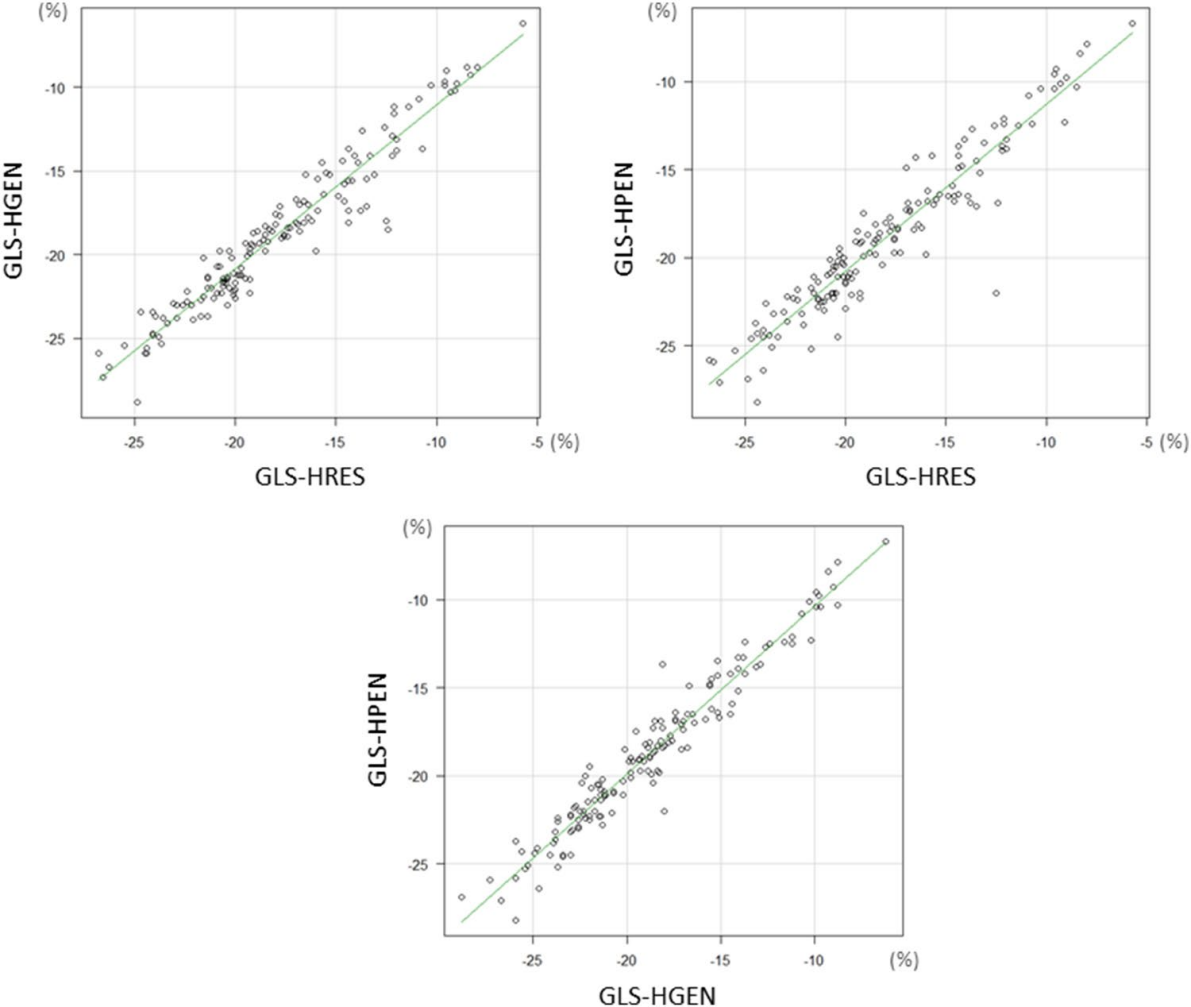
Correlation of global longitudinal strain among frequency presets. Correlation between GLP-HRES and GLS-HGEN (upper left), between GLS-HRES and GLS-HPEN (upper right) and between GLS-HGEN and GLS-HPEN (lower). GLS denotes global longitudinal strain; GLS-HRES, GLS-HRES, and GLS-HPEN, GLS measured by the HRES, HGEN and HPEN preset, respectively.

The mean value of GLS-HRES in the study patients was − 17.9 ± 4.4*%*, GLS-HGEN was − 18.8 ± 4.5% and GLS-HPEN was − 18.8 ± 4.4% (Table 1). There were good correlations among these GLS values (GLS-HRES vs. GLS-HGEN, r = 0.96 [95% CI 0.95 to 0.97], *p* < 0.0001; GLS-HRES versus GLS-HPEN, r = 0.95 [0.93 to 0.96], *p* < 0.0001; GLS-HGEN and GLS-HPEN, r = 0.97 [0.96 to 0.98], *p* < 0.0001) (Fig. 2).

**Table 1:** Global longitudinal strain and ultrasound transmit frequency.

	N	GLS-HRES	GLS-HGEN	GLS-HPEN	*p*
All patients	151	− 17.9 ± 4.4%	− 18.8 ± 4.5%^‡^	− 18.8 ± 4.4%^‡^	< 0.0001
Image quality					
Optimal	104	− 19.0 ± 4.1%	− 19.7 ± 4.2%^‡^	− 19.7 ± 4.2%^‡^	< 0.0001
Suboptimal	47	− 15.7 ± 4.4%	− 17.0 ± 4.5%^‡^	− 16.9 ± 4.2%^‡^	< 0.0001
Etiology					
Coronary artery disease	58	− 17.4 ± 4.3%	− 18.5 ± 4.4%^‡^	− 18.4 ± 4.6%^‡^	< 0.0001
Others	93	− 18.3 ± 4.5%	− 19.0 ± 4.5%^‡^	− 19.1 ± 4.3%^‡^	< 0.0001
LVEF					
< 50%	24	− 12.3 ± 3.4%	− 12.8 ± 3.7%*	− 12.8 ± 3.4%^†^	0.003
≥ 50%	127	− 19.0 ± 3.8%	− 20.0 ± 3.6%^‡^	− 19.9 ± 3.6%^‡^	< 0.0001

Continuous variables are expressed as mean ± standard deviation.

*GLS* global longitudinal strain, *GLS-HRES* GLS measured by HRES preset, *GLS-HGEN* GLS measured by HGEN preset, *GLS-HPEN* GLS measured by HPEN preset, *LVEF* left ventricular ejection fraction. *P* value for difference among 3 frequency presets.

*p < 0.05, ^†^*p* < 0.01, ^‡^*p* < 0.0001 versus GLS-HRES.

**Figure 3:**
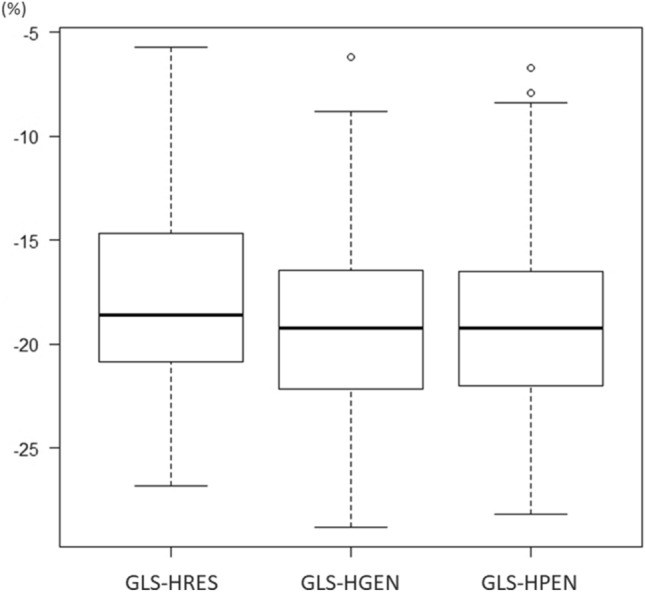
Difference in GLS among frequency presets. Box-and-whisker plots of global longitudinal strain (GLS) by 3 frequency presets. GLS-HRES, GLS-HRES, and GLS-HPEN denotes GLS measured by the HRES, HGEN and HPEN preset, respectively.

There was significant difference in GLS among three frequency presets (*p* < 0.0001), and the magnitude of GLS-HRES was significantly smaller than GLS-HGEN (*p* < 0.0001) and GLS-HPEN (*p* < 0.0001) (Table 1, Fig. 3). No difference was observed between GLS-HGEN and GLS-HPEN (*p* = 0.61). The absolute and relative differences between GLS-HRES and GLS-HGEN were 1.1 ± 1.0% and 6.9 ± 7.2%, respectively. Those between GLS-HRES and GLS-HPEN were 1.1 ± 1.2% and 7.1 ± 8.6%, respectively (Table 2). The absolute and relative differences between ultrasound settings were similar among IE33, Affiniti and EPIQ (Table 2).

**Table 2:** Absolute and relative difference in GLS among HRES and other presets.

	Absolute difference	*p*	Relative difference	*p*
*GLS-HRES versus GLS-HGEN*				
All patients	1.1 ± 1.0%		6.9 ± 7.2%	
Image quality				
Optimal	1.0 ± 0.7%	0.004	5.4 ± 4.0%	< 0.0001
Suboptimal	1.5 ± 1.4%		10.3 ± 10.7%	
Echo device				
IE33	1.2 ± 0.9%	0.60	6.7 ± 5.3	0.50
Affiniti	1.2 ± 1.1%		8.1 ± 8.8	
EPIQ	1.1 ± 1.0%		6.4 ± 7.2	
Etiology				
Coronary artery disease	1.3 ± 1.0%	0.20	7.6 ± 6.4%	0.33
Others	1.1 ± 1.0%		6.5 ± 7.6%	
LVEF				
< 50%	0.8 ± 0.6%	0.10	7.0 ± 4.4%	0.97
≥ 50%	1.2 ± 1.0%		6.9 ± 7.6%	
*GLS HRES versus GLS HPEN*				
All patients	1.1 ± 1.2%		7.1 ± 8.6%	
Image Quality				
Optimal	1.0 ± 0.9%	0.11	5.7 ± 4.8%	0.002
Suboptimal	1.4 ± 1.6%		10.2 ± 13.3%	
Echo device				
IE33	1.3 ± 1.0%	0.60	7.9 ± 7.4%	0.75
Affiniti	1.0 ± 1.0%		6.7 ± 7.7%	
EPIQ	1.1 ± 1.3%		6.7 ± 9.8%	
Etiology				
Coronary artery disease	1.1 ± 1.0%	0.86	6.4 ± 5.6%	0.48
Others	1.2 ± 1.3%		7.5 ± 10.1%	
LVEF				
< 50%	0.7 ± 0.7%	0.04	6.1 ± 6.3%	0.54
≥ 50%	1.2 ± 1.2%		7.3 ± 9.0%	

Continuous variables are expressed as mean ± standard deviation. Relative difference was calculated as [absolute difference/GLS-HRES] × 100 (%). The unit of absolute difference of GLS (%) was represented as ‘%’. P value for the difference between subgroups. GLS denotes global longitudinal strain; GLS-HRES, GLS-HGEN, GLS-HPEN, GLS measured by HRES-, HGEN- and HPEN preset, respectively; LVEF, left ventricular ejection fraction.

**Figure 4:**
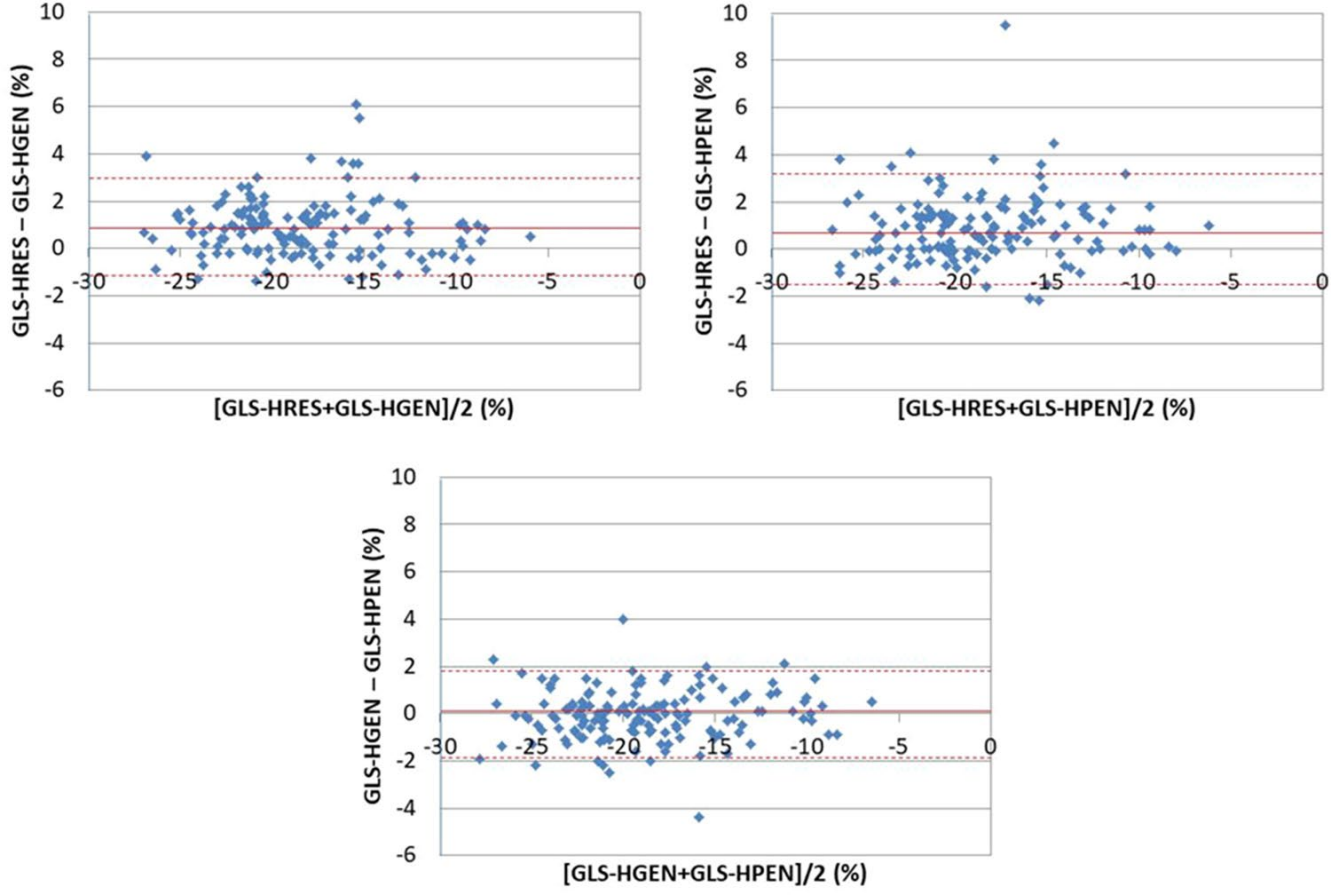
Bland–Altman plots for the difference of GLS among frequency presets. The bias is shown by the solid lines and the levels of agreement (1.95 × SD) by dotted lines.

Bland–Altman plots showed that there was no systematic difference between any pairs among 3 presets (GLS-HRES and GLS-HGEN, *p* = 0.57; GLS-HRES and GLS-HPEN, *p* = 0.82; GLS-HGEN and GLS-HPEN, *p* = 0.34) (Fig. 4).

### GLS and image quality

Image quality was suboptimal in 47 patients (31.1%). The rate of suboptimal image was 30.2% in IE33, 48.6% in Affiniti and 22.5% in EPIQ. There was a significant difference in its rate among 3 machines (*p* = 0.02), and EPIQ had significant lower rate of suboptimal images than Affiniti (*p* = 0.03). No difference was observed between IE33 and other two devices.

**Figure 5:**
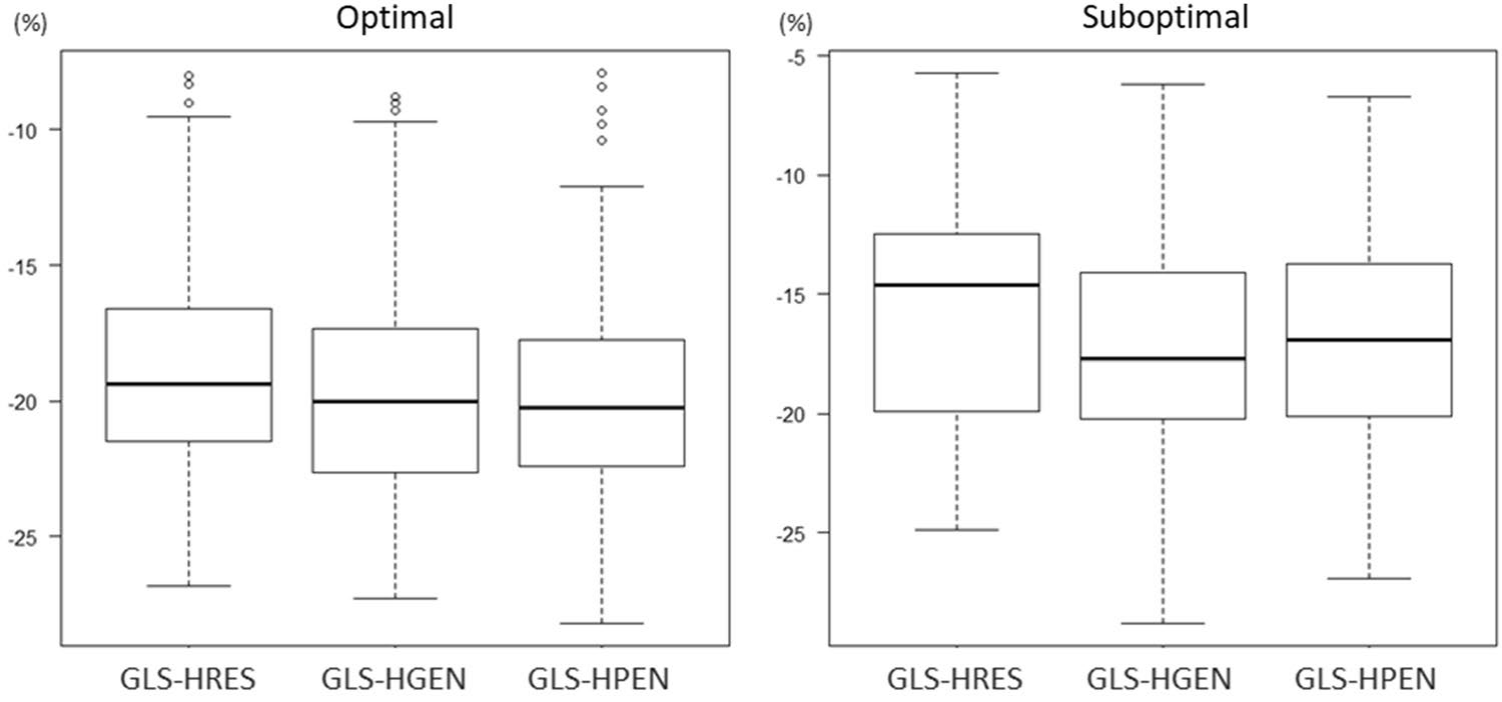
Difference in GLS and image quality. Box-and-whisker plots of GLS-HRES, GLS-HRES, and GLS-HPEN in 104 patients with optimal image quality (left) and in 47 patients with suboptimal quality (right). Difference in GLS among presets was observed in both subsets.

Significant difference in GLS among 3 presets was observed both in patients with optimal quality (*p* < 0.0001) and in those with suboptimal image quality (*p* < 0.0001) (Table 1, Fig. 5). GLS-HRES was significantly lower than GLS-HGEN and GLS-HPEN in both groups (Table 1). A significant interaction was observed between GLS variability by frequency and image quality (*p* = 0.013 by mixed design ANOVA). The absolute and relative difference between GLS-HRES and GLS-HGEN (1.5 ± 1.4% and 10.3 ± 10.7%, respectively) was larger in patients with suboptimal image quality than in those with optimal quality (1.0 ± 0.7%, *p* = 0.004, and 5.4 ± 4.0%, *p* < 0.0001). No difference was observed in absolute difference between GLS-HRES and GLS-HPEN between 2 subgroups (1.4 ± 1.6% vs. 1.0 ± 0.9%, *p* = 0.11), whereas the relative difference was significantly larger in patients with suboptimal image quality (10.2 ± 13.3% vs. 5.7 ± 4.8%, *p* = 0.002) (Table 2).

### GLS and ischemic etiology or ejection fraction

A significant difference in GLS among 3 presets was observed both in 58 patients with CAD (*p* < 0.0001) and in others (*p* < 0.001), and the magnitude of GLS-HRES was smaller than that of GLS-HGEN and GLS-HPEN in both groups (Table 1). No difference in LVEF was observed between patients with CAD and others (CAD, 59 ± 10%; others 61 ± 9%, *p* = 0.28). A significant difference in GLS was observed among 3 presets in 24 patients (15.9%) with impaired LVEF (*p* = 0.003) and in 127 patients with preserved LVEF (*p* < 0.0001). The magnitude of GLS-HRES was smaller than that of GLS-HGEN and GLS-HPEN in both groups (Table 1). There was no interaction between variation of GLS among presets and CAD category (*p* = 0.35 by mixed design ANOVA) or LVEF category (*p* = 0.24). No difference was observed in the absolute and relative differences in GLS among these subgroups, except the absolute difference between GLS-HGEN and GLS-HPEN in LVEF subgroups (Table 2).

## Discussion

The present study demonstrated that the magnitude of GLS obtained by higher frequency preset (GLS-HRES) was significantly smaller than that by lower frequency presets (GLS-HGEN and GLS-HPEN). The difference between GLS-HRES and other GLS was 1.1% as in absolute strain value and about 7% in relative difference. The difference in GLS among the frequency presets was interacted with image quality, although GLS variation was observed even in those with optimal image quality. To our knowledge, this is the first report that changes in transmit ultrasound frequency made small but significant difference in GLS by 2DSTE.

The present study had some unique features. We acquired each apical image by different frequency presets with as little interruption as possible, and a transducer was held carefully to avoid the change of position. Thus, we acquired 3 images on each apical image plane with minimal changes in recording condition except ultrasound frequency. We did not exclude patients with suboptimal images while reliable speckle tracking was obtained because adjustment of ultrasound frequency is usually required in these patients.

The GLS variation among 3 presets was interacted with the image quality of patients. The HRES preset, which had the lowest ultrasound penetration, provided lower magnitude of GLS than the others. Although a significant GLS variation was observed even in patients with optimal image quality, the absolute difference between GLS-HRES and GLS-HGEN was greater in patients with suboptimal image quality than in those with optimal quality. The ultrasound penetration may be the major cause of the GLS variation in the present study. Lower frequency achieves better ultrasound penetration which may lead to better tracking of myocardial speckles. The effect could be more preeminent in the suboptimal quality images, while those rated as good quality by visual method still could have potential for improvement.

Another potential mechanism involves changes in the ultrasound features of the acquired images. The "speckles" tracked by the 2DSTE technique originate from the interference of ultrasound waves with structures smaller than the ultrasound wavelength. The effect of changes in ultrasound frequency on the characteristics of these speckles remains unclear. Even if such changes are not visually apparent, they could affect the traceability of speckles in automated 2DSTE. Other modifications in ultrasound characteristics may also affect the measurement of GLS. The present results indicated that GLS measurement was slightly affected by transmit ultrasound frequency, but the difference was limited.

The absolute difference of 1% or relative difference of 5% in GLS can be ignored in most cases, and the present results confirmed that GLS can be reliably measured regardless of ultrasound frequency. However, the small difference may have clinical impact in some specific situations. After cancer treatments with anthracycline, trastuzumab or pertuzumab, a relative decrease in GLS of 10% to 15% is considered as the most sensitive parameter for predicting cardiotoxicity^31^. A relative decrease in GLS magnitude of > 15% from baseline is a strong indication of subclinical LV dysfunction associated with cancer therapy^32^. Relative difference caused by ultrasound frequency may lead to overestimation of GLS deterioration, which may lead to unnecessary interruption of cancer treatment^18,33^.

The present study had several limitations. We used a vendor-specific software package for the calculation of GLS. Variability of strain values may still exist between software packages provided by vendors^22,34^, although standardization of strain measurement among vendors has been addressed by the international committee^25^. The Q-station software used in the present study was not the newest version. It is unclear whether the present results can be generalized to other vendors’ software or vendor-independent software, which may use a different algorithm for speckle tracking, or to the newer version of the same vendor-specific software. As a single-center study with a limited number of patients, there may be a bias in patient selection. A multicenter, prospective study using a vendor-independent software is needed to definitively determine the effect of ultrasound frequency on GLS.

Although we were careful to keep conditions other than frequency as identical as possible in each image acquisition, slight changes in image plane or breath holding may occur. We consider that the possible change in image acquisition was too small to cause 1% absolute difference in GLS magnitude. We used 3 different ultrasound devices, which had different frequency setting under the same name, different beamforming technique and software. Although there was no difference in absolute and relative changes in GLS between 3 devices (Table 2), the possible effects of different devices on the present results could not be completely excluded. We only measured GLS in the present study, and it is unclear whether the effects of ultrasound frequency may differ among myocardial segments.

Instead of these limitations, the present study clarified the effect of ultrasound frequency on the GLS measurement. GLS can be reliably measured in most cases regardless of ultrasound frequency, though it may be interpreted carefully in some clinical settings.

## Data Availability

Data generated during the current study are available from Zenodo data repository as 10.5281/zenodo.8174001.
